# A Deep Learning Framework Integrating the Spectral and Spatial Features for Image-Assisted Medical Diagnostics

**DOI:** 10.1109/ACCESS.2021.3133338

**Published:** 2021-12-06

**Authors:** Susmita Ghosh, Swagatam Das, Rammohan Mallipeddi

**Affiliations:** Electronics and Communication Sciences UnitIndian Statistical Institute Kolkata 700108 India; Department of Artificial IntelligenceKyungpook National University 34986 Daegu 7027021 South Korea

**Keywords:** Medical imaging, diagnostic solution, COVID-19 detection, discrete cosine transform, discrete wavelet transform, deep learning, class imbalance, saliency map

## Abstract

The development of a computer-aided disease detection system to ease the long and arduous manual diagnostic process is an emerging research interest. Living through the recent outbreak of the COVID-19 virus, we propose a machine learning and computer vision algorithms-based automatic diagnostic solution for detecting the COVID-19 infection. Our proposed method applies to chest radiograph that uses readily available infrastructure. No studies in this direction have considered the spatial aspect of the medical images. This motivates us to investigate the role of spectral-domain information of medical images along with the spatial content towards improved disease detection ability. Successful integration of spatial and spectral features is demonstrated on the COVID-19 infection detection task. Our proposed method comprises three stages – Feature extraction, Dimensionality reduction via projection, and prediction. At first, images are transformed into spectral and spatio-spectral domains by using Discrete cosine transform (DCT) and Discrete Wavelet transform (DWT), two powerful image processing algorithms. Next, features from spatial, spectral, and spatio-spectral domains are projected into a lower dimension through the Convolutional Neural Network (CNN), and those three types of projected features are then fed to Multilayer Perceptron (MLP) for final prediction. The combination of the three types of features yielded superior performance than any of the features when used individually. This indicates the presence of complementary information in the spectral domain of the chest radiograph to characterize the considered medical condition. Moreover, saliency maps corresponding to classes representing different medical conditions demonstrate the reliability of the proposed method. The study is further extended to identify different medical conditions using diverse medical image datasets and shows the efficiency of leveraging the combined features. Altogether, the proposed method exhibits potential as a generalized and robust medical image-assisted diagnostic solution.

## I. Introduction

The proper diagnosis of any medical condition plays an important role in effective treatment and also in the prevention of any infectious disease to spread out. Various Machine learning-based diagnostic solution has been proposed to ease such a process of manual diagnosis that requires domain expertise and long training time [1]. The recent outbreak of Coronavirus disease 2019 (COVID-19) is the third significant Coronavirus outbreak in less than 20 years. In this context, a computer-based diagnostic solution with readily available infrastructure even in rural areas around the globe is the need of the hour. According to [2], a chest radiograph of a COVID-19 infected person exhibit ‘patchy or diffuse reticular–nodular opacities and consolidation, with basal, peripheral and bilateral predominance’. Thus, the readily and widely available infrastructure for X-rays may be utilized for primary and immediate assessment for detecting COVID-19 infection.

We aim to develop an automatic model using machine learning algorithms that would aid the clinician as an adjunct tool for the diagnosis of COVID-19 infection. We pose this COVID-19 detection problem as a three-class classification paradigm where the classes are *Normal*, *Pneumonia* (non-COVID), and *COVID-19* by utilizing deep learning algorithms. The reason behind choosing *Pneumonia* as one of the classes is that Pneumonia and COVID-19 can be easily confused with one another. Both non-COVID-19 Pneumonia and COVID-19 may exhibit ground glass patterns in the lung due to lung infiltration and consolidation. Over the last decade, deep learning, a subfield of machine learning has gained popularity in assisting as a diagnostic aid. The successful application of deep learning can be found in diagnostic assessment of different biomedical conditions such as arrhythmia detection [3], skin cancer classification [4], breast cancer detection [5], brain disease classification [6], Pneumonia detection from chest X-ray images [7] and lung segmentation [8]. These studies on the application of deep learning algorithms on medical imaging data show the efficiency of a deep learning algorithm in expressing complex patterns that are even difficult to capture in untrained eyes. These studies motivate us to exploit deep learning algorithms for characterizing such intricate, differentiating patterns to identify the underlying medical condition.

Few recent studies have endeavored in detecting COVID-19 infection from the chest X-ray images with the help of deep learning algorithms. Wang and Wong [9] has proposed a Convolutional Neural Network (CNN) based architecture build using generative synthesis, referred to as COVID-net which is trained on 13,975 CXR images across 13,870 persons belongs to the categories of *Normal*, *Pneumonia*, and *COVID-19* class. They have reported achieving an overall test accuracy of 93.3% with sensitivity and precision to *COVID-19* class of 91.0% and 98.9% respectively. In another study [10], authors have presented a two-stage network to classify four classes, namely *Normal*, *Bacterial*, *Tuberculosis* (TB), and *Viral Pneumonia/COVID-19*. They first trained an extended fully convolutional (FC)-DenseNet103 for image segmentation purposes, thereafter a patched-based CNN was trained by the segmented 403 lung images. The proposed method yields an accuracy of 88.9% with a specificity of 96.4% on the test data comprising 99 samples. Their study is further extended to three-class (*Normal*, *Pneumonia*, and *COVID-19*) classification which yielded an accuracy of 91.9%. Though, this study achieved high sensitivity for *COVID-19* class (100%), the low precision value (76.9%) for *COVID-19* class is not appropriate for any practical scenario. The DarkNet model was implemented using seven convolutional layers and various filterings on each layer for automatic detection of COVID-19 using the raw chest X-ray images [11]. The model aimed at providing correct diagnostic predictions for binary classification (COVID vs. No-Findings) and multi-class classification (COVID vs. No-Findings vs. *Pneumonia*). This system yielded a classification accuracy of 98.08% for binary classes and 87.02% for multi-class cases for a dataset of a limited number of samples. The performances of various neural architectures for detecting COVID-19 infection from chest X-rays were evaluated in [12]. Their results indicated that deep neural networks aided with X-ray imaging could detect prominent biomarkers pertinent to COVID-19 infection, while the best accuracy, sensitivity, and specificity obtained were reported as 96.78%, 98.66%, and 96.46% respectively. Another study [13] claimed the superiority of COVID-CAPS, a modeling framework based on Capsule Networks with fewer trainable parameters over CNN-based models for COVID-19 detection. COVID-CAPS obtained an Accuracy of 95.7%, Sensitivity of 90%, Specificity of 95.8%, and Area Under the Curve (AUC) of 0.97 for binary classifications. Another study [14] proposed CNN model with end-to-end training process for classifying among the chest x-ray images of *Normal* and *COVID-19* classes. Integration of deep learning-based features extractor with Support Vector Machine (SVM) based classifier has achieved an accuracy of 92.6%. However, the binary classification performances were reported on very limited samples. Togaçar et. al. [15] employed the Fuzzy Color technique for preprocessing the chest X-ray images followed by an image stacking operation to eliminate the existing noises. The integration of deep learning architectures with SVM classifier led to an accuracy of 99.27% for three-class (*Normal*, *Pneumonia* and *COVID-19*) classification. However, the proposed method was validated on a dataset of a total of 458 chest radiograph images. Another similar study [16] showed that the fusion of five deep learning models via integration stacking achieved 99.08% accuracy on limited samples.

The body of literature related to COVID-19 infection identification from chest X-rays shows the efficient application of deep learning algorithms. Among the above-mentioned studies, few studies have achieved excellent performances, yet, validation on a larger dataset is necessary. All the mentioned studies have investigated the spatial domain characteristics of chest x-rays. To the best of our knowledge, no studies on COVID-19 from chest X-rays have reported investigating the spectral characteristics of the same. This motivates us to investigate the unexplored spectral aspect of chest x-ray towards the COVID-19 infection detection. We aim to validate the hypothesis of the presence of complementary information in the spectral and spatial domain of chest x-ray that will improve disease detection ability. We employ two popular tools for transforming images in spectral and spectral as well as spatial domains namely, Discrete Cosine Transform (DCT) and Discrete Wavelet Transform (DWT) to study the spatio-spectral characteristics of the chest X-ray images. The idea behind the usage of DCT and DWT transformation is to capture any information which is complementary to spatial information and might aid in discriminating among the three classes considered in this study. The DCT decomposes the image into several spectral sub-bands with cosine function as a basis function, whereas, DWT has the advantage of assimilating both spatial and spectral domains simultaneously. Hence, chest radiograph images are studied in three different domains concurrently, namely, pixel, DCT, and DWT to bring about the potential of each of the fields in characterizing the considered medical conditions. The patterns that characterize different medical conditions are complex and intricate. State-of-the-art CNN shows notable efficiency to model complex patterns in the domain of image classification. Hence, we employ ResNet50, a state-of-the-art CNN architecture to extract features from the pixel, DCT, and DWT domain images. The features extracted in these three domains are integrated and the final class prediction is performed using a multilayer perceptron (MLP) network. The detailed description of our proposed method is given in Section II. Medical imaging datasets often have a limited number of samples for comparatively uncommon disease classes. This class imbalance usually directs to inferior performances corresponding to the minority class. Relevant techniques such as class weight, unbiased validation performance metrics, has been employed to prevent such undesired outcome and are discussed in Section II in detail.

Furthermore, we extend our experiment to six other medical imaging datasets. These datasets incorporate a variety in types of imaging techniques, number as well as types of diseases to be detected, and class imbalance. The purpose of this extended study is to validate the hypothesis of the presence of complementary information in the spectral and spatial domain of medical images. This study overall indicates the generalization ability of the proposed method towards a diagnostic solution using medical images.

The contributions of this study are listed below.

• We propose an automatic computer vision and machine learning-based diagnostic solution for medical images developed on the complementary knowledge of the spatial and spectral domain.
• The proposed method is validated by carrying out experiments on eight diverse medical imaging datasets suggesting its robustness and capability of generalization.
• The classification performances along with saliency maps demonstrate the fusion of spatial, spectral, and spatio-spectral domain features enhances the disease detection capability of the classification model.
• Analyzing the classification performance on the COVID-19 dataset reveals that the performance of the proposed method is unbiased to age and gender factors.

The rest of the paper is structured as follows. Along with the detailed description of the datasets, the methodology used in this study is presented in Section II. The experimental results and relevant discussion is given in Section III. Section IV concludes our study along with the possible future directions.

## II. Methods and Materials

### A. Dataset Details

**FIGURE 1.**
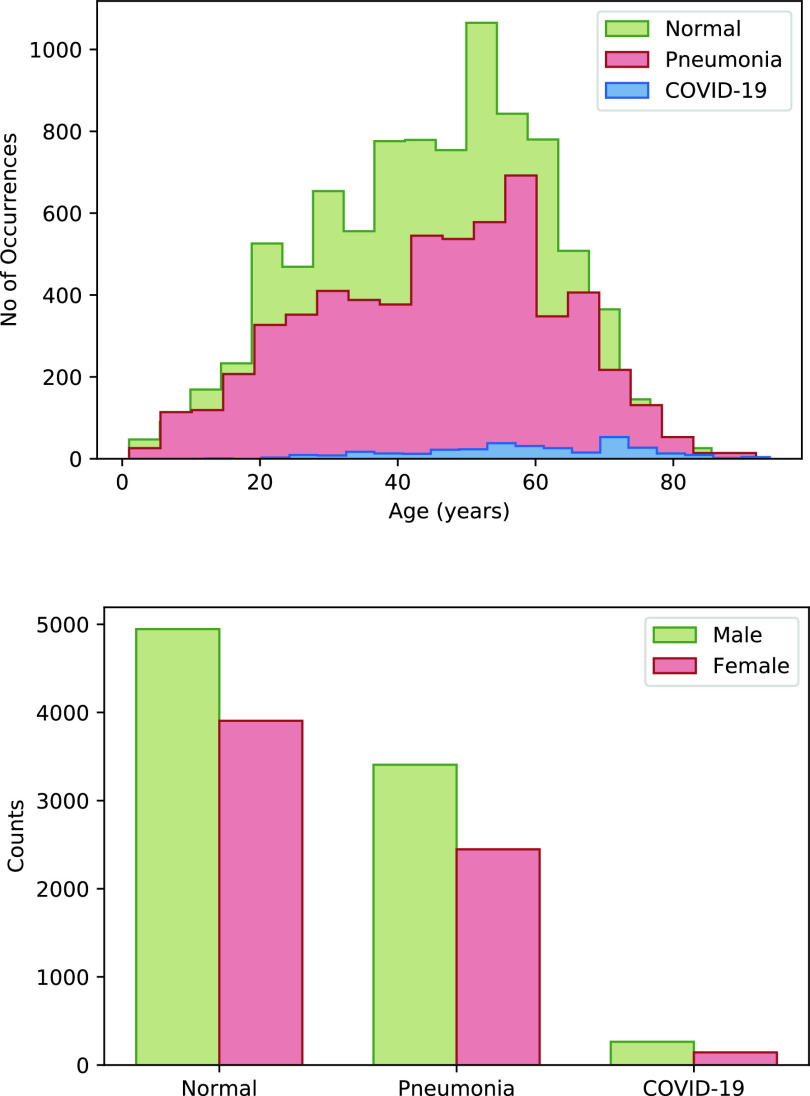
Age and gender distribution of persons belong to *Normal*, *Pneumonia* and *COVID-19* classes are presented in upper and lower panel of the figure respectively.

We validate our hypothesis primarily on a dataset comprising 15476 chest x-ray images from 15279 persons which belong to any three of the categories representing three types of medical conditions namely — *Normal*, *Pneumonia* and *COVID-19*. Images belongs to *COVID-19* category has been taken from these four sources [17]–[20] whereas, samples of *Pneumonia* and *Normal* class been collected from these sources [19], [21]. This dataset is referred COVID-19 dataset. Collecting samples from more than one source leads to diversity in the nature of the images. Thus, the experimental results on this dataset are robust and close to a practical scenario. The number of samples and the number of patients that belong to each of the three classes are stated in Table. 1. Among the 15279 patients considered in this study, the ages and gender of 15029 and 15112 patients are known respectively. The age distribution of the subjects in each category and gender distribution in each class is shown in Figure. 1. The dataset suffers from the problem of class imbalance. The imbalance ratio ( $\rho $) defined by the ratio of the number of samples in the majority class to that of the minority class is 15.45. The chest X-ray images are of different sizes, thus in the preprocessing step, we resized it to the size of $224\times 224 \times 3$ where the third dimension represents the number of channels. The results reported in this study are yielded by five-fold cross-validation. Out of the five folds, one is kept for testing, another for validation purposes while the rest of the folds are used to train the model. While partitioning the data into five-folds, it is always ensured that samples from one patient are never groped into two or more folds to maintain the integrity of the reported result.

**TABLE 1:** Number of Samples and Patients Belonging to Normal, Pneumonia and COVID-19 Class

	Number of Samples	Number of Patients
Normal	8851	8851
Pneumonia	6052	6034
COVID-19	573	394
Total	15476	15279

Additionally, seven medical imaging datasets of different modalities e.g. X-ray, histopathology, mammography, etc, and medical conditions are also used for the validation of the hypothesis. The diversity in the number of classes i.e. the medical conditions, image modalities, and the imbalance ratio makes the experiment more robust. A detailed description of each of the datasets used in this study is presented in Tab. 2. We have used a subset of these original CBIS-DDSM [22], DR [23] and BHI [24] datasets as they comprise a large number of samples. Each dataset is split into training and testing samples maintaining an 80:20 ratio, except for Chestxray1 [9] where 100 test samples are provided for each of the three classes.

**TABLE 2:** Detailed Description of Medical Imaging Datasets Used in This Study

Dataset	Type of data	Disease Type	Number of classes	Number of Training samples	Imbalance ratio
CBIS-DDSM [22]	Breast mammography images	Abnormality in breast tissue	2	8941	6.55
DR [23]	Eye images	Diabetic Retinopathy	2	13052	1.33
Colorectal Histology [25]	Histology images	Colorectal cancer tissue type	8	4000	1
ISIC18 [26]	Dermoscopic images of skin	Skin disease	7	8166	58.86
Chestxray1 [9]	Chest x-ray images	COVID-19	3	13898	16.84
Chestxray2 [27]	Chest x-ray images	Pneumonia	2	5232	2.88
BHI [24]	Breast Histopathology images	Invasive Ductal Carcinoma	2	12952	2.79

### B. Discrete Cosine Transform

The Discrete Cosine Transform (DCT) of an image is a real transformation that transforms the image from spatial domain to frequency domain by linear combinations of weighted basis functions pertinent to its frequency components [28]. DCT of an image $X$ of dimension $N \times N$ is given by the following equation.

\begin{align*}&\hspace {-0.5pc}DCT_{x}(u,v) = \frac {2}{N}C(u)C(v)\sum _{m = 0}^{N-1}\sum _{n = 0}^{N-1}X(m,n) \\&\times cos\left[{\frac {\pi (2m+1)u}{2N}}\right]cos\left[{\frac {\pi (2n+1)v}{2N}}\right],\tag{1}\end{align*}

where, $X(m,n)$ denotes the pixel value $X$ in $(m,n)$ coordinate, $u =0,\ldots,N-1$, $v = 0,\ldots,N-1$ and $\begin{aligned} C(k) = \begin{cases} \frac {1}{\sqrt {2}}\quad if \,\,k = 0, \\ 1 \quad otherwise. \end{cases} \end{aligned}$

In this study, images are of dimension $224\times 224 \times 3$. DCT is applied to the images by considering each channel separately over the segmented patches of size $8 \times 8$. Thus, the dimension of the DCT images is the same as the input images.

### C. Discrete Wavelet Transform

The discrete wavelet transform (DWT) uses multiresolution filter banks to perform the wavelet analysis [29]. It represents the signal in terms of the wavelet coefficients from which it is possible to reconstruct the original signal once again. The signal is represented in various frequency bands by the wavelet coefficients. There can be several ways to process these coefficients, thus, endowing DWT with attractive properties over linear filtering.

The DWT is applied to the image $X$ of size $(N\times N)$ to achieve four decomposed subband images of $(N/2 \times N/2)$ dimension. The process includes application of a set of half-band low pass and high pass filters to the rows of the image and followed by the decimation by a factor of 2. The same procedure is applied to the two subband images obtained in the previous step but this time along the columns. Thus, it results in decomposed images in four different frequency bands which can be mathematically expressed as follows,

\begin{align*} LL(x,y)=&\sum _{m}\sum _{n}h_{0}(m-2x)h_{0}(n-2y)X(m,n), \tag{2}\\ HL(x,y)=&\sum _{m}\sum _{n}h_{0}(m-2x)h_{1}(n-2y)X(m,n), \tag{3}\\ LH(x,y)=&\sum _{m}\sum _{n}h_{1}(m-2x)h_{0}(n-2y)X(m,n), \tag{4}\\ HH(x,y)=&\sum _{m}\sum _{n}h_{1}(m-2x)h_{1}(n-2y)X(m,n).\tag{5}\end{align*}

**FIGURE 2.**
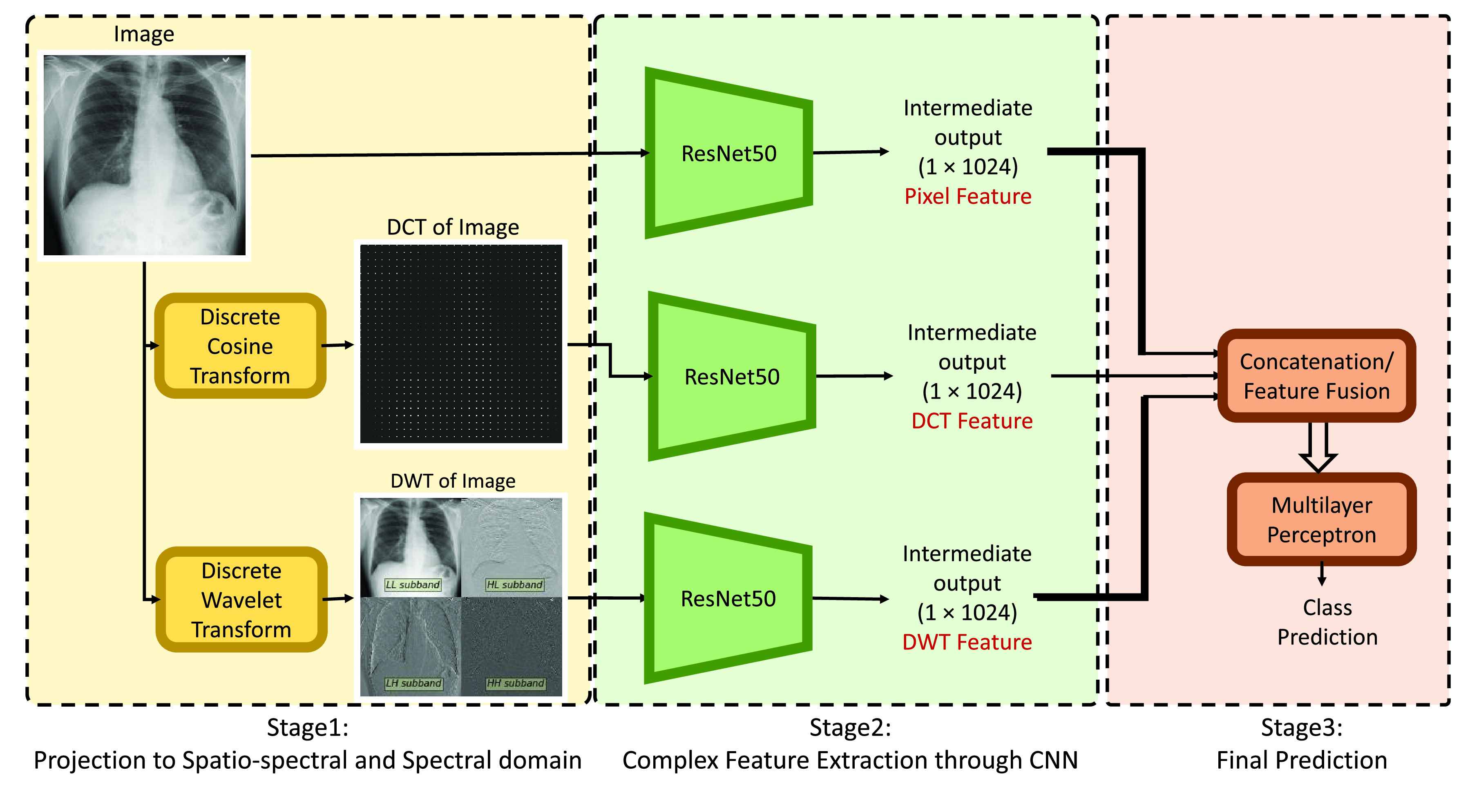
Schematic diagram of the classification framework.

Here, $h_{0}$ and $h_{1}$ are half band low and high pass filters respectively. Thus, $LL$ subband approximates the image i.e. low-frequency content of the image, whereas, three other bands i.e. $LH$, $HL$, and $HH$ subbands contain the details i.e. the high-frequency content of the image. The four subband images of size $N/2 \times N/2$ are arranged in the manner as shown in Figure 2 to form one image of size $N\times N$. Similar to DCT, DWT is also applied individually on each of the channels of images and the transformed image dimension is also the same as the input image dimension.

### D. Performance Metrics

Let, $\textbf {C}$ be the confusion matrix for n-class classification where element $C_{ij}$ indicate the number of samples of $i^{th}$ class predicted as of $j^{th}$ class. The following metrics presented in terms of the elements of the confusion matrix are used to quantify the classification capability of machine learning models.

\begin{align*} Sensitivity_{i}=&\dfrac {C_{ii}}{\sum _{j}C_{ij}}, \tag{6}\\ Precision_{i}=&\frac {C_{ii}}{\sum _{j}C_{ji}}, \tag{7}\\ F1{-}score_{i}=&\frac {2*Sensitivity_{i}*Precision_{i}}{(Sensitivity_{i}+Precision_{i})}.\tag{8}\end{align*}

Along with the above-mentioned performance metrics, three other measurements are also used to quantify the performances of models — average class-specific accuracy/sensitivity (ACSA), average class-specific precision (ACSP), and an average class-specific F1-score (ACSF) respectively indicating sensitivity, precision, and F1-score averaged over all the classes, thus imparting equal priority to all the classes. Hence, these metrics quantify the model performances without being influenced by the existing class imbalance. The mean absolute deviation of the F1-score (MADF) is also reported along with ACSF to quantify the spread of individual class wise F1-scores.

\begin{equation*} MADF = \frac {1}{n}\sum _{i}|F1{-}score_{i}- \frac {1}{n}\sum _{i}F1{-}score_{i}|.\tag{9}\end{equation*}

### E. Machine Learning Framework

The block diagram presented in Figure 2 shows an outline of the complete framework for the proposed diagnostic system. The proposed system consists of three stages — projection by using DCT and DWT, complex feature extraction, and prediction. In the first stage, the images are projected to spectral as well as spatio-spectral domain by the application of DCT and DWT respectively as described in Section II-B and Section II-C respectively. Hence, at the end of this stage, we get three types of images of equal dimension in three different domains — pixel, DCT, and DWT (shown in Figure 2).

The objective of the second stage is to extract the features from output images of the previous stage. The underlying pattern that distinguishes one medical condition from another is intricate in nature. Convolutional Neural Network (CNN) can extract complex features from input images. ResNet50 is one of the widely used CNN architectures in the domain of image categorization. Thus, we choose to employ ResNet50 to serve the purpose of extracting complex features. Moreover, the reasonable number of trainable parameters of ResNet50 makes it suitable for medical image classification tasks where there are limited training samples available. Three separate ResNet50 models are trained for three different types of images. The output feature is extracted from an intermediate layer of trained ResNet50 models which results in complex features of dimension 1024. For training each of the ResNet50 models, the categorical cross-entropy loss function is optimized with the Adam optimization algorithm with a learning rate of 0.0001 for 300 epochs with a batch size of 32. The model with the best validation ACSA (defined in Section II-D) is chosen for feature mapping. ACSA is chosen for this purpose as this measure is unbiased to class imbalance present in the training data.

The third stage comprises feature level fusion followed by final prediction using a classification head. Three types of feature vectors, each of dimension 1024 are combined in all seven possible combinations by the concatenation operation. The dimension of the concatenated feature vector is presented in Table 3. The resulting feature vector is fed to a classification head for final prediction. The Multilayer Perceptron (MLP) is chosen as the classification head because of its efficiency to model complicated nonlinear relationship between input and output vectors. The MLP network comprises one input layer, two hidden layers with 256 and 64 nodes, and one output layer. The leaky ReLU activation function is applied after each layer except the last layer which uses softmax as the activation function. To train the MLP network, the mean square error loss function is optimized with the Adam optimization algorithm with a learning rate of 0.0002 for 300 epochs with a batch size of 32. The model with the best training ACSA is evaluated with the test data.

**TABLE 3:** All Possible Combination of Pixel, DCT and DWT Features and Corresponding Integrated Feature Vector’s Dimension

Combination Type	Dimension of Feature Vector
pixel	$1\times 1024$
DCT	$1\times 1024$
DWT	$1\times 1024$
pixel+DCT	$1\times 2048$
pixel+DWT	$1\times 2048$
DCT+DWT	$1\times 2048$
pixel+DCT+DT	$1\times 3072$

The effect of class imbalance present in the datasets is handled by incorporating the penalty factor of class weights computed as $N/(c*N_{i})$ to the loss function while training the ResNet50 model and MLP model, where $c$ is the number of classes, $N$ and $N_{i}$ is the number of total samples and the number of samples that belong to $i^{th}$ class respectively.

All the experiments (Python scripts) are executed using Keras with TensorFlow as backend on a computer with Intel core i5 processor running at 2.40 GHz using 16 GB of RAM and NVIDIA GeForce RTX 2060 GPU with 6 GB RAM.

## III. Experimental Results and Discussion

In this section, we present the results obtained by applying our proposed method to the medical imaging datasets.

### A. Result on COVID-19 Dataset

Due to the severity of the COVID-19 outbreak, the primary focus is paid on the elaborated study that is conducted COVID-19 dataset (described in Section I). The result obtained from the above-mentioned experiment (Section II-C) is presented in Table 4. The classification performances are quantified by class-wise sensitivity, precision, and F1-score. Additionally, we also present sensitivity, precision, and F1-score averaged over the different classes to measure overall performance. They are mentioned as ACSA, ACSP, and ACSF, and these metrics are defined in Section II-D. Table 4 compares the performance of the seven feature combinations as listed in Table 3. By comparing the performances of three types of features (pixel, DCT, and DWT) when used individually, pixel and DWT feature yields better results than DCT features. However, the fusion of any two types of features shows greater performance than any of the features while used solely for classification. Furthermore, the best classification performance is achieved when all three types of features i.e. pixel, DCT, and DWT are combined altogether (pixel+DCT+DWT). The corresponding value of ASCA, ACSP, ACSF are 93.78%, 91.30% and 92.42% respectively. It is important to note that the above-mentioned feature combination also yields the best sensitivity to the COVID-19 class which is 95.28% as we aim to detect COVID-19 infection with sensitivity as high as possible. These observations suggest the presence of complementary information in pixel, DCT, and DWT features. To validate the above-said statement from a statistical perspective, the Wilcoxon rank-sum test is performed on the ACSA, ACSP, and ACSF values. Table 5 summarises the result obtained from tests, where the null hypothesis is that the performance of three features combined together (pixel+DCT+DWT) is equivalent to that of other combinations of the features. The null hypothesis is rejected when the p-value is less than 0.05, confirming that the samples belong to different distributions whereas a larger p-value suggests that the two distributions are similar. Furthermore, if test-statistics comes out to be negative with a p-value less than 0.05, we infer that the performance of pixel+DCT+DWT is superior to that of the other feature combination considered in the test. Table 5 shows that the test-statistics is never positive indicating performance of pixel+DCT+DWT is superior (in most of the cases, marked by ${}^{*}$) or comparable (in a few cases) with the performance of the rest of the feature combinations. Thus, the outcome indicates that both DCT and DWT features capture information complementary to that of pixel feature that enhances the discriminating capability among the three classes considered in this study. Hence, our hypothesis of the existence of complementary information in the spatial and spectral domain of medical images is validated for the COVID-19 dataset.

**TABLE 4:** The Classification Performances All Possible Combination of Pixel, DCT and DWT Features in Detection of Normal, Pneumonia and COVID-19 Classes are Presented. Performances are Quantified by Sensitivity, Precision. Average Class Specific Sensitivity, Specificity and F1-Score (ACSA, ACSP, and ACSF) are Also Reported. All the Performance Metrics are Reported on 5-Fold Cross-Validation. Comparing Among Different Feature Combination, Best Value of Each Metrics are Marked in Bold

Feature Combinations	Sensitivity(%)			ACSA	Precision(%)			ACSP	F1-Score(%)			ACSF
	Normal	Pneumonia	COVID-19		Normal	Pneumonia	COVID-19		Normal	Pneumonia	COVID-19	
Pixel	94.55	90.26	92.60	92.47	93.89	92.04	84.78	90.24	94.21	91.14	88.44	91.26
	(±0.94)	(±0.65)	(±03.59)	(±01.49)	(±0.53)	(±01.25)	(±03.1)	(±01.4)	(±0.55)	(±0.75)	(±02.79)	(±01.30)
DCT	93.15	87.21	80.93	87.10	91.66	89.40	81.18	87.41	92.39	88.27	80.82	87.16
	(±0.70)	(±01.72)	(±04.58)	(±01.21)	(±0.69)	(±0.96)	(±04.86)	(±01.6)	(±0.42)	(±0.68)	(±01.81)	(±0.76)
DWT	95.06	89.75	92.15	92.32	93.51	92.79	84.93	90.41	94.28	91.24	88.30	91.27
	(±0.43)	(±0.98)	(±02.38)	(±0.88)	(±0.52)	(±0.56)	(±05.20)	(±01.72)	(±0.39)	(±0.63)	(±03.12)	(±01.14)
Pixel+DCT	95.18	90.27	93.25	92.90	93.93	92.91	85.34	90.72	94.55	91.56	89.02	91.71
	(±0.56)	(±0.96)	(±03.41)	(±01.19)	(±0.56)	(±0.74)	(±03.65)	(±01.44)	(±0.49)	(±0.81)	(±02.41)	(±01.06)
Pixel+DWT	95.15	90.91	94.86	93.64	94.30	93.04	86.39	91.24	94.72	91.96	90.32	92.33
	(±0.99)	(±0.64)	(±02.75)	(±0.94)	(±0.44)	(±01.23)	(±04.91)	(±01.86)	(±0.58)	(±0.85)	(±02.47)	(±01.08)
DCT+DWT	95.31	89.72	93.03	92.69	93.51	93.04	86.45	91.00	94.40	91.34	89.58	91.77
	(±0.55)	(±01.38)	(±01.89)	(±0.63)	(±0.82)	(±0.71)	(±03.21)	(±01.29)	(±0.52)	(±0.87)	(±02.25)	(±0.93)
Pixel+DCT+DWT	95.49	90.58	95.28	93.78	94.13	93.51	86.26	91.30	94.80	92.01	90.45	92.42
	(±0.85)	(±0.80)	(±2.52)	(±0.78)	(±0.49)	(±1.08)	(±4.12)	(±1.62)	(±0.53)	(±0.85)	(±1.74)	(±0.86)

**TABLE 5:** Statistics and p-Value Obtain in Wilcoxon Rank-Sum Test With Performance of Pixel+DCT+DWT Feature Combination is Compared With That of the Rest of the Feature Combinations. The Statistics Indicating Superiority of Pixel+DCT+DWT Feature Combinations are Marked With *

	ACSA		ACSP		ACSF	
	Statistics	p-value	Statistics	p-value	Statistics	p-value
Pixel	$-5.04^{*}$	< 0.05	$-2.53^{*}$	< 0.05	$-4.37^{*}$	< 0.05
DCT	$-8.61^{*}$	< 0.05	$-2.82^{*}$	< 0.05	$-8.62^{*}$	< 0.05
DWT	$-7.05^{*}$	< 0.05	−0.25	0.80	$-4.82^{*}$	< 0.05
Pixel-DCT	$-4.55^{*}$	< 0.05	−1.09	0.27	$-2.67^{*}$	< 0.05
Pixel-DWT	−0.12	.90	$-8.50^{*}$	< 0.05	−0.68	0.5
DCT-DWT	$-6.38^{*}$	< 0.05	−0.62	0.53	$-3.03^{*}$	< 0.05

The number of the hidden layers and the number of nodes in hidden layers of the MLP network are similar for all the combination of features types. However, the number of nodes in the input layers varies with the type of feature integration. For example, the number of nodes in the input layer of MLP is 1024 for pixel, DCT, DWT features, 2048 for Pixel+DCT, pixel+DWT, and DCT+DWT feature combination, and 3072 for pixel+DCT+DWT feature combination. Consequently, the number of trainable parameters also increases with the number of nodes in the input layers. Thus, the question may arise that whether the superior performance of the combined pixel+DCT+DWT features is due to the integration of the features or the increased number of trainable parameters of the model. In search for the answer, we performed another experiment, where the considered architecture of the model is the same as it is used for pixel+DCT+DWT features. We construct the new feature vector for pixel by concatenating the pixel feature thrice so that the dimension of the new pixel feature vector becomes $1 \times 3072$ vector. A similar operation is applied to DCT and DWT features also. The performances of these new feature vectors are evaluated on the above-mentioned MLP individually. Table 6 compares these performances with pixel+DCT+DWT features (last row of Table 4). The test-statistics obtained from Wilcoxon rank-sum tests confirm the superiority of the performance of combined features concerning that of single features where the model complexity is kept unchanged.

**TABLE 6:** The Performances of the New Pixel, DCT, and DWT Features Evaluated on the MLP Model That Has Similar Architecture as Used in the Case of Piexel+DCT+DWT Features. The New Pixel, DCT, and DWT Feature Vectors are Constructed by Concatenating Each Type of Feature Three Times

	Sensitivity(%)			ACSA	Precision(%)			ACSP	F1-Score(%)			ACSF
Feature	Normal	Pneumonia	COVID-19		Normal	Pneumonia	COVID-19		Normal	Pneumonia	COVID-19	
Pixel	94.84	90.10	93.65	92.86	93.96	92.66	81.85	89.49	94.39	91.35	87.30	91.02
	(±1.22)	(±0.54)	(±3.80)	(±1.54)	(±0.23)	(±1.81)	(±4.17)	(±1.69)	(±0.61)	(±0.80)	(±3.28)	(±1.47)
DCT	93.08	86.91	83.64	87.88	91.72	89.65	77.69	86.35	92.38	88.24	80.25	86.96
	(±0.72)	(±1.69)	(±3.55)	(±0.84)	(±0.58)	(±1.08)	(±7.11)	(±2.34)	(±0.41)	(±0.66)	(±2.92)	(±1.22)
DWT	94.86	90.01	92.42	92.43	93.77	92.62	83.31	89.90	94.30	91.28	87.53	91.04
	(±0.81)	(±1.23)	(±2.82)	(±1.06)	(±0.65)	(±1.01)	(±5.76)	(±1.9)	(±0.42)	(±0.68)	(±3.81)	(±1.37)
Pixel+DCT+DWT	95.49	90.58	95.28	93.78	94.13	93.51	86.26	91.30	94.80	92.01	90.45	92.42
	(±0.85)	(±0.80)	(±2.52)	(±0.78)	(±0.49)	(±1.08)	(±4.12)	(±1.62)	(±0.53)	(±0.85)	(±1.74)	(±0.86)

#### 1) Interpreting Classification Model

**FIGURE 3.**
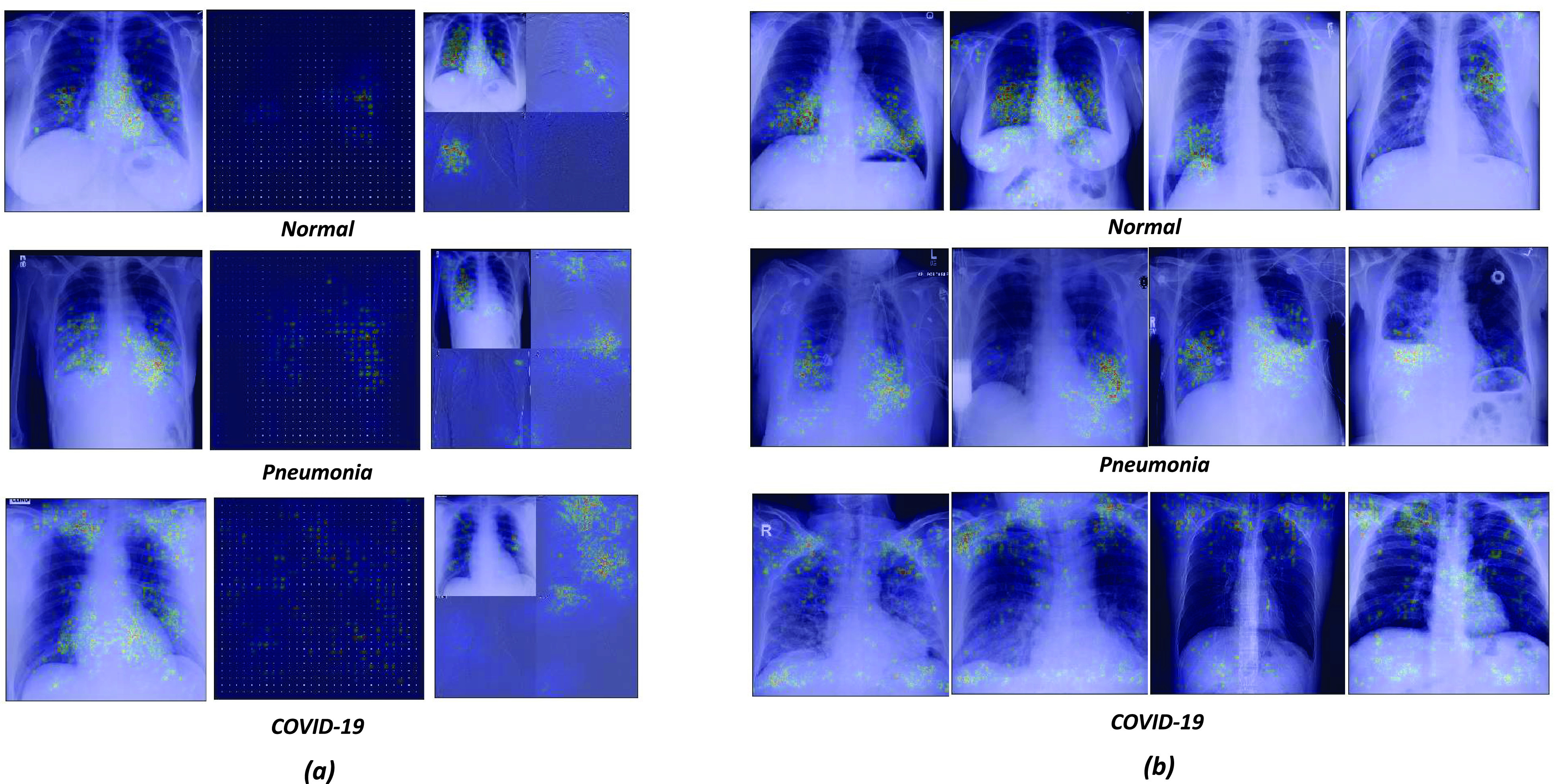
(a) Saliency maps corresponding to *Normal*, *Pneumonia* and *COVID-19* classes are presented for chest X-ray image(left panel), DCT image (middle panel) and DWT image (right panel). The highlighted regions play important role in characterizing the three classes. (b) Saliency maps of chest X-ray images sampled down from three classes are presented.

In this section, we attempt to understand how information from DCT and DWT domains contributes to enhancing the discriminative potential of the model. For this purpose, the saliency map i.e. the gradient of the class activation function concerning the input images is visualized. Thus, the saliency map for a particular class quantifies the amount of change in classification score caused by the small change in image pixel [30] indicating the decisive regions in the image. In Figure 3(a), the saliency map produced by the proposed model is shown for one image and its corresponding DCT and DWT images from each class. It confirms that all three types of images contribute to decoding the classes. Moreover, the saliency map of our model for each class validates its reliability as the highlighted regions from each of the images lie in the lung and its surrounding area. It is also noticed that the subband of DWT that represents the higher frequency component of the images does not contribute significantly towards disease detection.

Moreover, we inspect the saliency map of chest X-ray images to learn if there is any apparent pattern corresponding to each class and correlate them with existing literature in the medical domain. Four such maps from each class are presented in Figure 3(b). While, saliency map of *Normal* class shows a wide variety in the area of chest X-ray that is highlighted, in case of *Pneumonia* class, the influential pixel cluster around the lower lobes of the both or either lung. On the other hand, along with the other lung regions, the upper lobe (bilateral) area of the lung is found to be persistently dominating in the case of *COVID-19* class. The bilateral Ground-glass opacities (GGO) have been reported in COVID-19 chest x-ray whereas the unilateral and central distribution of GGO has been found in chest x-ray of Pneumonia patients [11]. This characteristics are consistent with the patterns shown the saliency maps of *COVID-19* and *Pneumonia* classes.

#### 2) Effect of Age and Gender on Classification Performance

**FIGURE 4.**
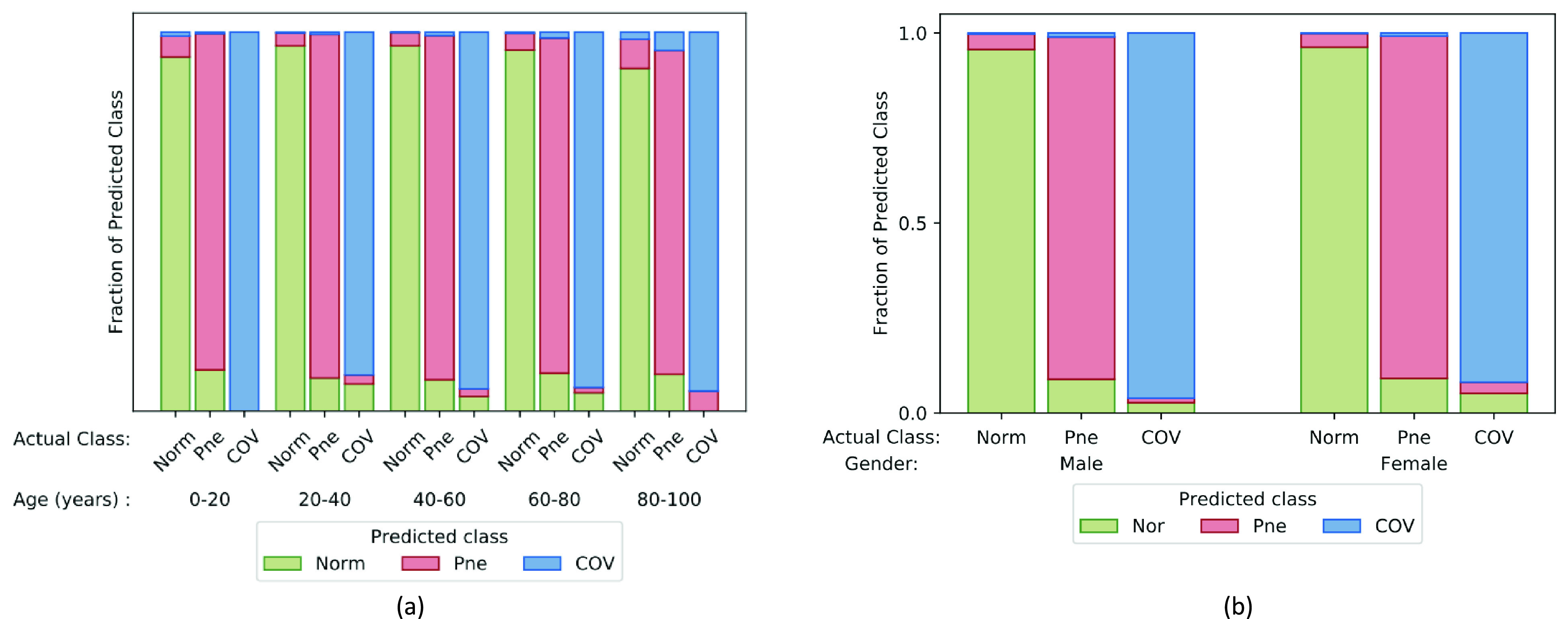
Age wise and gender wise classification performance is shown in (a) and (b) respectively. *Norm*, *Pne*, and *COVD-* represent *Normal*, *Pneumonia*, and *COVID-19* respectively. The height of the three stacked up bars indicate the fraction of a particular class that belongs to three classes. For instance, the green, red and blue bars in the leftmost bar of panel (a) indicate the fraction of *Normal* samples (of 0–20 years age group) that is classified as *Normal*, *Pneumonia* and *COVID-19* class.

The ages of the patients considered in this study varied from as low as 1 year to as high as 94 years Figure 1. To analyze if there is any biasedness of age on the classification performance, we carry out an analysis by clustering samples into five age groups – 0–20 years, 20–40 years, 40–60 years, 60–80 years, and 80–100 years. For each class, we investigated how the performance of the classifiers varies over different age groups. Figure 4(b) depicts the fraction of samples of a particular class classified as *Normal*, *Pneumonia*, and *COVID-19* classes. It is observed that over different age groups, the proposed method yields a similar trend suggesting that the age factor does not influence our classification results.

Furthermore, another similar analysis is executed to investigate the effect of gender of the patients on the classification result. The outcome of the analysis is shown in Figure 4(b) which demonstrates that the gender of the patients does not behave as a factor in classification performance. All the classification performances, reported in this section are yielded using the combined features (i.e. pixel, DCT, and DWT).

#### 3) Comparison With COVID-Net

We compare our result with the state-of-the-art experimental result reported in [9]. For this purpose, we apply our methodology to the same dataset as mentioned in [9]. The comparison in terms of sensitivity, precision, and F1-score is presented in table 7. The main aim of this study is to detect the COVID-19 class with as high sensitivity as possible and also maintain reasonably high precision at the same time. Though the value of the average class-specific accuracy, precision, and F1-score obtained in the proposed method is comparable with the result presented in [9], however, the higher sensitivity to *COVID-19* class of the trained model makes our proposed method more suitable for practical implementations.

**TABLE 7:** Comparison of Performance of Proposed Method With COVID-Net ([9])

	Sensitivity(%)			ACSA	Precision(%)			ACSP	F1-Score(%)			ACSF
	Normal	Pneumonia	COVID-19		Normal	Pneumonia	COVID-19		Normal	Pneumonia	COVID-19	
COVID-net	95.00	94.00	91.00	93.33	90.50	91.30	98.90	93.57	92.70	92.63	94.79	93.37
Proposed	94.80	91.80	94.30	93.63	91.60	92.55	96.92	93.69	93.17	92.17	95.59	93.64

### B. Result on Additional Datasets

We validate our proposed method on other medical imaging datasets as described in section I. These datasets comprise different modalities, medical conditions as well as class imbalance ratios. Since the number of classes varies in the range from two to eight, instead of showing class-specific metrics, we present average class-specific metrics. Additionally, the mean absolute deviation of the F1-score (MADF) is used to quantify the spread of the individual class-wise F1-score. The lower the value of MADF indicates lower dispersion among the F1-score of each class. Table 8 presents the classification performance for seven datasets listed in Table 2 for Pixel, DCT and DWT features individually. The efficiency of these features is also tested by performing feature level fusion (FF) and also score level fusion (SF). The best result obtained in feature level fusion or score level fusion is reported in Table 8. It is observed that the combination of two or three types of features among pixel, DCT, and DWT has surpassed the result obtained while using the each of features separately. Moreover, the lower MADF with higher ASCF of combined features also indicates the superiority in handling class imbalance. Thus, the result obtained in this experiment supports our hypothesis of the presence of complementary information in spatial and spectral domains in medical images that assist in decoding the underlying medical condition.

**TABLE 8:** The Performances of Pixel, DCT, DWT, and Pixel+DCT+DWT in Identifying Different Medical Condition for Different Datasets as Listed in Table 2

	Pixel				DCT				DWT					Combined			
Dataset	ACSA	ASCP	ASCF	MADF	ACSA	ASCP	ASCF	MADF	ACSA	ASCP	ASCF	MADF	Combination Type	ACSA	ASCP	ASCF	MADF
BHI	89.32	90.44	89.85	5.09	87.15	87.00	87.07	6.33	85.52	86.47	85.97	7.02	pixel+DCT+DWT, SF	89.86	90.98	90.40	4.81
	(±0.53)	(±0.44)	(±0.28)	(±0.18)	(±0.51)	(±0.34)	(±0.21)	(±0.18)	(±0.45)	(±0.57)	(±0.33)	(±0.18)		(±0.3)	(±0.23)	(±0.17)	(±0.1)
CBIS_DDSM	91.19	94.59	92.79	5.36	77.58	80.46	78.82	15.78	86.60	87.58	87.05	9.51	pixel+DCT+DWT, FF	91.98	94.17	93.00	5.17
	(±0.52)	(±0.3)	(±0.26)	(±0.21)	(±1.55)	(±1.42)	(±0.43)	(±0.53)	(±0.89)	(±0.82)	(±0.26)	(±0.23)		(±1.24)	(±1.14)	(±0.48)	(±0.37)
CH	95.28	95.42	95.32	3.34	92.36	92.38	92.33	5.07	94.05	94.03	94.02	4.04	pixel+DWT, FF	95.63	95.67	95.62	2.96
	(±0.34)	(±0.25)	(±0.32)	(±0.33)	(±0.42)	(±0.29)	(±0.36)	(±0.42)	(±0.2)	(±0.19)	(±0.2)	(±0.28)		(±0.24)	(±0.21)	(±0.23)	(±0.24)
CHESTXRAY1	89.97	90.31	90.00	1.04	87.33	88.30	87.34	0.82	91.23	91.37	90.02	91.95	pixel+DCT+DWT, FF	93.63	93.69	93.64	1.44
	(±0.46)	(±0.42)	(±0.45)	(±0.21)	(±0.47)	(±0.38)	(±0.49)	(±0.24)	(±0.45)	(±0.46)	(±0.45)	(±0.22)		(±0.43)	(±0.42)	(±0.43)	(±0.34)
CHESTXRAY2	71.62	86.31	72.87	12.44	68.90	85.41	69.58	14.57	74.46	88.00	76.18	10.49	pixel+DWT, SF	74.68	88.08	76.42	10.35
	(±1.86)	(±0.58)	(±2.17)	(±1.37)	(±1.28)	(±0.37)	(±1.44)	(±0.89)	(±1.35)	(±0.4)	(±1.51)	(±0.93)		(±1.28)	(±0.37)	(±1.44)	(±0.89)
DR	69.62	69.42	69.49	4.24	60.17	60.28	60.14	6.67	65.02	64.79	64.73	4.19	pixel+DCT, FF	70.08	69.76	69.84	3.80
	(±0.41)	(±0.35)	(±0.36)	(±0.3)	(±0.48)	(±0.35)	(±0.45)	(±1.51)	(±0.77)	(±0.58)	(±0.67)	(±1.3)		(±0.68)	(±0.63)	(±0.64)	(±0.38)
ISIC18	75.89	76.40	75.86	9.49	62.70	66.97	64.51	13.93	63.44	65.81	64.03	15.17	pixel+DCT+D WT, FF	78.53	77.55	77.69	9.69
	(±0.63)	(±1.53)	(±0.87)	(±0.44)	(±0.97)	(±0.89)	(±0.76)	(±0.82)	(±1.99)	(±1.36)	(±1.21)	(±0.74)		(±1.38)	(±1.35)	(±0.79)	(±0.75)

## IV. Conclusion and Future Directions

In this study, we present a novel medical image-based diagnostic solution for the detection of various underlying medical conditions. Along with the spatial information of the medical images, we exploited the less explored spectral-domain information of the medical images. We aspire to achieve an enhancement in disease detection performance by integrating the features from spatial, spectral, and spatio-spectral space. Our proposed system comprises three stages — conversion of a spatial image into the spectral and spatio-spectral domains, feature mapping from higher to lower dimension using CNN architecture, and final classification using MLP. The potential and the robustness of the proposed method are demonstrated by using eight diverse medical imaging datasets of different modalities, diseases to be identified, and class imbalance ratio.

The results suggest that the spatial, spectral and spatio-spectral domain features are solely capable of representing distinguishing characteristics of the underlying medical conditions. However, integration of those three types of features results in a significant increment in classification performance, suggesting that all three types of features possess complementary information. The saliency maps also validate the integrity of our proposed model. A detailed study on COVID-19 shows that the performance of our method is unbiased to gender and age factor. In comparison to the method proposed in [9] for COVID-19 detection, our method achieved significantly higher sensitivity to *COVID-19* class and also marginal improvement in average class-specific accuracy, precision, and F1-score. Altogether, our study demonstrates a novel approach in identifying various diseases using the medical images that can assist the healthcare worker in the primary screening process.

This study can be extended to other types of available medical images for generalization purposes. The implementation of other deep learning algorithms for feature extraction purposes can be explored towards the enhancement of the performance. Three separate ResNet50 networks are trained for extracting features from pixel, DCT, and DWT images. As a future research direction of this study, we consider overcoming this limitation. We have used an iterative training approach for updating the parameters of both CNN and MLP networks. The adaptation of non-iterative learning mechanism as used in Neural Networks with Random Weights (NNRW) [31]–[33], semi-random learning mechanism as used in Bidirectional stochastic configuration network (BSCN) [34] for updating the weights of the models should reduce the training time significantly. It will be interesting to study how the performance of the proposed method will be influenced by such non-iterative training. Future research direction may consider the interesting aspect of non-iterative training.
